# Physical Activity In Renal Disease (PAIRED) and the effect on hypertension: study protocol for a randomized controlled trial

**DOI:** 10.1186/s13063-019-3235-5

**Published:** 2019-02-08

**Authors:** Stephanie Thompson, Natasha Wiebe, Gabor Gyenes, Rachelle Davies, Jeyasundar Radhakrishnan, Michelle Graham

**Affiliations:** 1 Division of Nephrology and Immunology, 11-112R CSB, 152 University Campus NW, University of Alberta, 11-112 Clinical Sciences Building, Edmonton, AB T6G 2G3 Canada; 2 Department of Cardiology, Mackenzie Health Science Centre, 8440-112 Street, Edmonton, AB T6G 2B7 Canada; 3 Innovation Physical Therapy, 15823 97 Street NW #209, Edmonton, AB T5X 0C7 Canada

**Keywords:** Exercise, Hypertension, Blood-pressure, Chronic kidney disease, Randomized trial, Ambulatory blood pressure

## Abstract

**Background:**

The prevalence of hypertension among people with chronic kidney disease is high with over 60% of people not attaining recommended targets despite taking multiple medications. Given the health and economic implications of hypertension, additional strategies are needed. Exercise is an effective strategy for reducing blood pressure in the general population; however, it is not known whether exercise would have a comparable benefit in people with moderate to advanced chronic kidney disease and hypertension.

**Methods:**

This is a parallel-arm trial of adults with hypertension (systolic blood pressure greater than 130 mmHg) and an estimated glomerular filtration rate of 15–45 ml/min 1.73 m^2^. A total of 160 participants will be randomized, with stratification for estimated glomerular filtration rate, to a 24-week, aerobic-based exercise intervention or enhanced usual care. The primary outcome is the difference in 24-h ambulatory systolic blood pressure after 8 weeks of exercise training. Secondary outcomes at 8 and 24 weeks include: other measurements of blood pressure, aortic stiffness (pulse-wave velocity), change in the Defined Daily Dose of anti-hypertensive drugs, medication adherence, markers of cardiovascular risk, physical fitness (cardiopulmonary exercise testing), 7-day accelerometry, quality of life, and adverse events. The effect of exercise on renal function will be evaluated in an exploratory analysis. The intervention is a thrice-weekly, moderate-intensity aerobic exercise supplemented with isometric resistance exercise delivered in two phases. Phase 1: supervised, facility-based, weekly and home-based sessions (8 weeks). Phase 2: home-based sessions (16 weeks).

**Discussion:**

To our knowledge, this study is the first trial designed to provide a precise estimate of the effect of exercise on blood pressure in people with moderate to severe CKD and hypertension. The findings from this study should address a significant knowledge gap in hypertension management in CKD and inform the design of a larger study on the effect of exercise on CKD progression.

**Trial registration:**

ClinicalTrials.gov, ID: NCT03551119. Registered on 11 June 2018.

**Electronic supplementary material:**

The online version of this article (10.1186/s13063-019-3235-5) contains supplementary material, which is available to authorized users.

## Background

In people with chronic kidney disease (CKD), hypertension is an important modifiable risk factor for both cardiovascular (CV) events and progressive renal dysfunction [1–5]. Delaying the progression of CKD is important because it enables patients to live longer without significant complications from CKD (malnutrition, bone and electrolyte disorders, anemia, higher risk of CV events) and the need for renal replacement therapy. Despite the significance of attaining blood pressure (BP) targets, the estimated prevalence of hypertension among clinical populations with CKD remains high at 67–86% [6, 7]. This is despite the use of multiple anti-hypertensive drugs and access to dietary counseling on low sodium intake (more than 90% of Canadian CKD clinics have a dietician on staff) [8].

Regular exercise is effective in reducing BP in people without CKD. BP reduction with regular aerobic exercise ranges from − 3.5 to − 6.1 mmHg/− 2.5 to − 3.0 mmHg [9–11]. Although guidelines for the management of CKD recommend exercise for CV health, randomized trials in people with impaired estimated glomerular filtration rate (eGFR) and hypertension are limited [12, 13]. In addition, compared to those with normal kidney function, people with CKD are more likely to need multiple anti-hypertensive drugs to attain BP targets [14, 15], suggesting that responsiveness to other approaches may also be reduced. As a result of this knowledge gap, exercise resources are not offered in the routine multidisciplinary care of people with CKD and the prevalence of sedentary behavior remains double that of the general population [8, 16].

The primary objective of this trial is to determine the efficacy of an 8-week, aerobic-based exercise intervention on systolic blood pressure (SBP) in people with moderate to severe CKD. We hypothesize that participants allocated to the exercise intervention will achieve a significant mean reduction in SBP as measured by the “gold standard,” 24-h ambulatory blood pressure (ABPM) compared to control. Results from this efficacy trial will also be used to inform the design of a larger, multi-center trial aimed at evaluating the effect of exercise on the risk of CKD progression.

### Objectives

#### Primary objective

The primary aim of this study is to determine the efficacy of an 8-week exercise intervention on SBP in people with moderate to severe CKD.

#### Secondary objective

The secondary objective is to evaluate the effect of exercise on other measures of BP: aortic stiffness, changes in the required dose of anti-hypertensive drugs, markers of CV risk, cardiorespiratory fitness, a physical activity, quality of life (QoL), and adverse events (AEs) at 8 and 24 weeks.

#### Tertiary objective

The tertiary objective is to inform the design of a larger, multi-center, randomized controlled trial (RCT) examining the effect of exercise on the progression of CKD; specifically, to establish trial processes, feasibility, and to estimate the variance of the treatment effect for eGFR.

## Methods

### Design, setting, and participants

**Fig. 1:**
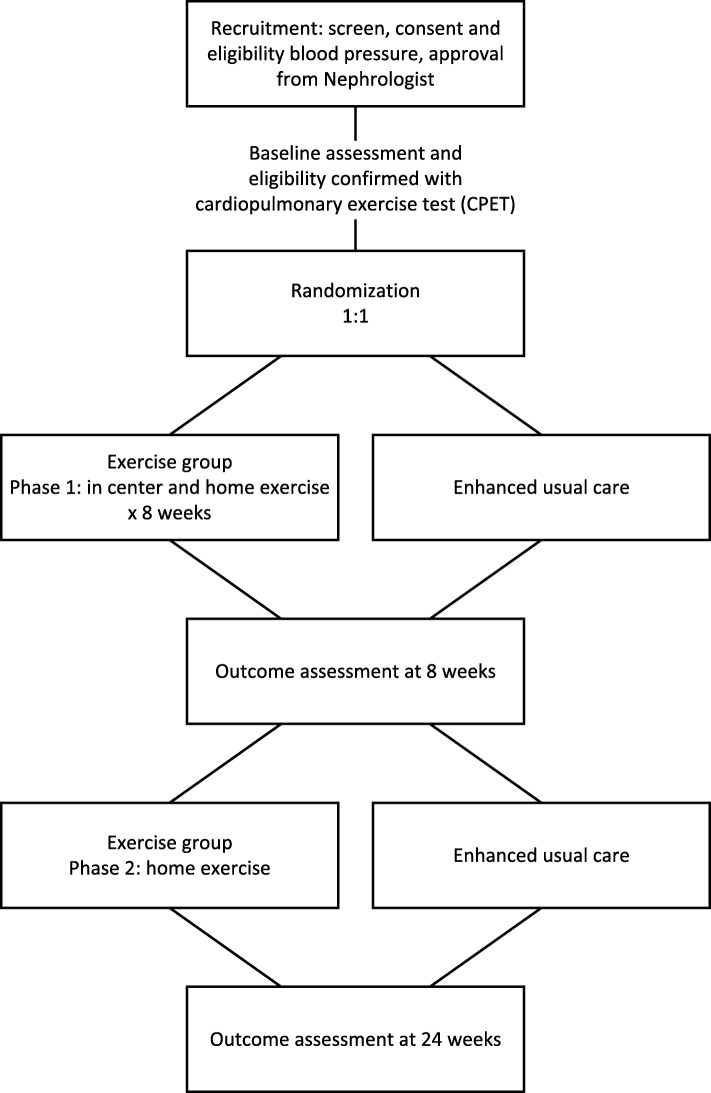
Flow of participants through the trial

The flow of participants through the trial is summarized in Fig. 1. Participants will be randomized to either an aerobic-based exercise intervention or a control. Participants will be recruited from both academic and community-based clinics within Alberta Kidney Care (AKC) North. AKC North is a single, provincially funded renal program in Edmonton, AB, Canada. The University of Alberta Research Ethics Board approved this protocol (Pro00078564). The study was prospectively registered (NCT0355119). The Standard Protocol Items: Recommendations for Interventional Trials (SPIRIT) Checklist is available as an additional document (Additional file 1).

### Recruitment and eligibility

A range of recruitment strategies will be employed, including direct recruitment from AKC North CKD clinics by study staff, posters, advertising through the local branch of The Kidney Foundation, and referrals from physicians and renal clinic staff. All recruitment materials and participant information has been co-developed or reviewed with a panel of patient advisors. Prior to eligibility screening, informed written consent will be obtained by the trial study coordinator from the potential participant.

#### Inclusion

Eligible patients are those aged 18 years or older, with hypertension, who are followed in an Edmonton-based CKD clinic with an eGFR between 15 and 44 ml/min per 1.73m^2^ on two occasions in the past year. Hypertension is defined as a resting SBP greater than 130 mmHg on screening measurement with a validated, electronic sphygmomanometer (Omron HEM-907XL (Omron Healthcare, Kyoto, Japan)), using the mean of three readings after discarding the first reading and at least one previous SBP measurement above 130 mmHg on a separate occasion within the past 6 months (home or clinic measurement). Although there is evidence that lower SBPs are beneficial, there is still debate regarding the optimal BP target in people with CKD [17, 18] and a target SBP of less than 130 mmHg is consistent with current Canadian CKD Management Guidelines [19]. Additional inclusion criteria are: able to ambulate with or without an assistive device for at least three consecutive minutes, capable of providing informed consent, English language sufficient to understand written information and comply with the testing and interventions, and approval of the attending nephrologist.

#### Exclusion

Resting SBP greater than 160 mmHg or resting DBP greater than 110 mmHg on screening BP clinic measurement, changes to BP medications within the past 8 weeks, arm circumference greater than 54 cm (size limit of large ABPM cuff), recent (within 6 weeks) or planned (within 6 months) major CV events or procedures, any absolute contraindication to exercise (American College of Sports Medicine Guidelines) [20], pregnant or planning to become pregnant within the next 9 months, life expectancy or predicted time to renal replacement therapy less than 12 months (attending physician judgment), planned move or hospital admission within the next 9 months, currently enrolled in an interventional clinical trial or a structured exercise program, significant barriers to participation (e.g., no available transportation to the training facility), solid organ transplantation, taking other medications known to affect BP (prednisone/cyclosporine) with expected adjustment in the next 9 months. Patients will be excluded from the study if exercise testing results preclude safe exercise training as defined by the American College of Cardiology/American Heart Association (ACC/AHA) guidelines [21], e.g., abnormal BP response, early ischemic changes, and unexpected life-threatening arrhythmia. The trial cardiologists will review the electrocardiogram at peak exercise on all participants.

### Randomization and blinding

Participants will be randomized (1:1) to enhanced usual care (measurement of physical activity levels) or the exercise intervention. A randomized permuted block design (of 4 and 6) will be used. To optimize comparability of the two groups at baseline, randomization will be stratified by baseline eGFR (15–29 ml/min/1.73m^2^ and 30–44 ml/min/1.73m^2^). The randomization sequence will be computer generated using Stata/MP 15.1 (https://www.stata.com/) and allocation will be concealed by web-based central randomization using the Research Electronic Data Capture System (REDCap 8.8.2© 2018 Vanderbilt University). Due to the nature of the intervention, participants cannot be blinded to group assignment; however, the primary outcome is objectively measured (24-h ABPM).

### Intervention arms

#### Exercise group

All participants will receive standard care according to the Canadian Guidelines for the Management of Chronic Kidney Disease [19]. Those allocated to the intervention group will participate in a 24-week exercise program delivered in two phases. Phase 1 is an 8-week program of once weekly, supervised, facility-based exercise sessions and twice-weekly, home-based sessions. The weekly, facility-based sessions will take place in a rehabilitation facility at the University of Alberta Hospital. Phase 2 is a 16-week, home-based exercise program, overseen by a kinesiologist. During this phase, participants will be progressed through their home-based exercise program from phase 1 on an individualized basis. In phase 2, participants are seen once by the kinesiologist, 4 months after study start (in the middle of the 16-week home phase) and as needed. The exercise intervention is primarily aerobic, as the BP-reducing effect of exercise is greater with aerobic versus dynamic resistance exercise in people with hypertension: − 8.3 (95% CI − 10.7, − 6.0)/5.2 (95% CI − 6.8, − 3.4) mmHg versus 0.47 (95% CI − 4.4, 5.3)/− 1.0 (95% CI − 3.9, 1.9) mmHg [9]. The mode of aerobic exercise (e.g., walking, cycling) will be individualized based on participant preference and feasibility for home. In both phases, participants will be prescribed exercise on a minimum of 3 days per week at a moderate intensity (50–60% heart rate reserve) based on the most recent cardiopulmonary exercise test (CPET). Participants will progress over phase 1 to accumulate 150 min of exercise per week [22]. Sessions may be continuous or interspersed as 10-min bouts [23–25]. In both phases, aerobic exercise will be supplemented with isometric resistance exercises (sustained muscle contraction with no change in muscle length). In both hyper- and normotensive populations, isometric resistance exercise is associated with change in SBP of − 6.8 (95% CI, − 7.9 to − 5.6) mmHg [26]. Isometric exercise is also feasible for the home setting and has shown to be safe in people with hypertension using the appropriate technique [27]. Isometric exercises will focus on large muscle groups. The period of time and the postures will be individualized and progressed over the course of the study period.

#### Control group (enhanced usual care)

Renal healthcare providers will continue their usual practice for all trial participants. Participants in the control group will also perform 7-day accelerometry. This is enhanced usual care because physical activity measurement is not routinely performed in CKD clinics. Accelerometry has been associated with increased levels of physical activity; however, this effect is transient and the health impact of activity monitoring is unclear [28]. Furthermore, this approach is critical for recruitment and retention in the control arm and to determine whether participants in the control arm increase their levels of physical activity. The control-arm participants will only receive their accelerometry data after they have completed the study.

### Adherence

To maximize adherence to exercise in the intervention group, we will apply the core set of determinants for behavior change from Bandura’s Social Cognitive Theory: knowledge of health benefits, self-efficacy (expectations about the ability to perform the behavior successfully) and outcome expectations (perceived consequences of the behavior) [29]. Strategies to increase self-efficacy for physical activity will be evidence-based (i.e., vicarious experience and formalized comparative feedback) [30]. The Self-efficacy for Exercise (SEE) scale will be used to evaluate participants’ self-efficacy expectations of exercise expectations [31]. Throughout the trial, the kinesiologist will use motivational interviewing techniques to identify and reinforce the participant’s goals and reasons for changing behavior. To assess adherence and to provide additional motivation, the trial kinesiologist will phone intervention participants every 2 weeks in phase 2. To promote retention, all participants who complete the trial will be offered an additional counseling session with the study kinesiologist and an activity-tracking device. These incentives are intended to assist control-arm participants with increasing their levels of physical activity. For members of the intervention group, these incentives are intended to support the maintenance of physical activity levels.

### Variables and their measurement

#### Baseline clinical information and study visits

A summary of the study schedule is shown in Table 1. Clinical data including cause of CKD, comorbidities, and medications will be collected via participant interviews, chart reviews, and reviews of clinical databases at the baseline study visit. Follow-up study visits are scheduled at 8 and 24-weeks. All participants will be contacted by phone every 2 weeks during the first 8 weeks of the trial to collect data on unscheduled visits and phone calls to healthcare providers, AEs, and changes to anti-hypertensive drugs. Over weeks 8 to 24, participants will be phoned every 4 weeks for this information. Data on AEs and medication changes will be verified using the electronic health repository.

**Table 1:** Overview of trial outcomes by study visit

Outcome measure	Method	Baseline	Study visit 1 8 weeks	Study visit 3 24 weeks
Blood pressure	24-h ABPM Clinic BP with an automated oscillometric device	X	X	X
Anti-hypertensive dosing	Tabulated using the Defined Daily Dose (DDD)	X	X	X
Aortic stiffness	Pulse-wave velocity	X	X	X
Medication adherence	Questionnaire	X	X	X
Markers of cardiovascular risk Lipids, HgA_1_C, C-reactive protein Albuminuria, sodium intake Body composition	Blood samples Spot urine sample Bioimpedance spectroscopy, BMI	X	X	X
Cardiorespiratory fitness	Cardiopulmonary exercise testing Six-min Walk Test	X	X	X
Physical activity	7-day accelerometry IPAQ-SF	X	X	X
Quality of life	KDQoL-36 EQ-5D	X	X	X
Self-efficacy	Self-efficacy for Exercise scale	X	X	X
Adherence	7-day accelerometry Participant logbook		X	X
Adverse events	Self report and health record verification	X	X	X

Baseline data is collected prior to randomization. Adverse events are also collected by phone follow-up every 2 weeks for the first 8 weeks and every 4 weeks from weeks 8 to 24

Abbreviations: *ABPM* ambulatory blood pressure monitoring, *BMI* Body Mass Index, *BP* blood pressure, *EQ-5D* EuroQol Health Questionnaire, *HgA*_*1*_*C* hemoglobin A_1_C, *IPAQ-SF* International Physical Activity Questionnaire Short Form, *KDQoL-36* The Kidney Disease and Quality of Life Instrument*,*

#### Primary outcomes

The primary outcome for the trial is the difference in 24-h ambulatory SBP after 8 weeks of exercise training compared to control. SBP is a stronger predictor of end-stage renal disease than DBP or other measures of arterial stiffness in people with CKD [1–3, 32]. Twenty-four-hour ABPM is the gold-standard method for BP measurement and more accurately predicts renal and CV risk in people with CKD than clinic BP [33]. Participants will be advised to perform their regular daily routine (but not to exercise) during the 24-h ABPM period or 24 h before the study visit (for clinic BP measurements). ABPM will be measured using a validated device (OnTrak Ambulatory Blood Pressure Monitor 90,227–1; Spacelabs Healthcare, Mississauga, ON, Canada) [34] worn on the non-dominant arm for a 24-h period. Readings will be obtained at 20-min intervals from 6 a.m. to 10 p.m. and at 30-min intervals from 10 p.m. to 6 a.m. according to a recommended protocol [35].

#### Secondary outcomes

##### Additional blood pressure and vascular outcomes

The difference in overall, daytime, nighttime ABPM, and clinic BPs at 8 and 24 weeks will be reported. Clinic BPs will be measured using the mean of three readings (after discarding the first reading), each 1 min apart. Arterial stiffness will be estimated using the pulse-wave velocity (PWV) between the (1) carotid and femoral and the (2) carotid and radial arteries with the Complior device. Recommendations for the conditions and measurement of PWV will be followed [36]. Mean PWV will be calculated as the average of at least 10 consecutive beats in order to cover a full respiratory cycle.

##### Changes in anti-hypertensive drugs

The Defined Daily Dose (DDD) of anti-hypertensive drugs will be used to calculate any changes to BP medications during the study [37]. Medication lists will be collected from the participant and verified with the pharmacy record (available through an electronic provincial data repository) at each study visit. At study visits, a brief questionnaire designed to capture both intentional and non-intentional medication adherence will be administered [38].

##### Markers of cardiovascular risk

Body Mass Index (BMI), C-reactive protein, hemoglobin A_1_C (HgA_1_C) (only participants with diabetes) and lipid profile. Urine studies include spot urinary sodium, protein, and creatinine. All samples will be collected according to routine laboratory protocol on a non-exercise day after an overnight fast. To measure fat-free mass, whole-body bioimpedance measurements will be performed using the Body Composition Monitor (BCM; Fresenius Medical Care, Germany).

##### Cardiorespiratory fitness

Peak VO_2_ (volume of oxygen consumption) using a calibrated TrueOne 2400 metabolic cart (ParvoMedics TrueOne, Murray, UT, USA) from the CPET will be used to evaluate exercise-training adaptations and to determine the dose of exercise that was performed. The preferred modality for the CPET will be a stationary bicycle. After 5 min of rest, participants will start cycling at 10 W with an increasing stepped workload of 10–20 W every 2 min. The CPET will be symptom-limited, with the aim of achieving a respiratory exchange ratio > 1.10 and a Borg Rating of Perceived Exertion greater than 8 on a 0–10 scale. VO_2_ will be defined as the highest oxygen uptake value recorded during the test. VO_2_ peak will be derived from breath-by-breath indirect calorimetry and recorded as the peak 20-s average VO_2_ during the final minute of exercise. Using the post-CPET heart rate recovery at 1 and 5 min, we will also explore the parasympathetic response to exercise. Other measures of functional capacity in the exercise group include the 6-min Walk Test [39], performed three times during phase 1 and once at the end of phase 2.

##### Physical activity and adherence

Accelerometry (Actigraph® GT3X+ Actigraph, LLC, Pensacola, FL, USA) will be used to measure the overall difference in physical activity levels between groups and adherence in the intervention group. Participants will be instructed to wear the Actigraph device around the waist for at least 10 waking hours for seven consecutive days. Best practice and research recommendations on the management and analysis of physical activity data will be followed [40]. In phase 1, adherence will be measured as the proportion of people who attended all eight in-center sessions within 12 weeks. For the home components of phase 1 and phase 2, adherence will be assessed as the proportion of exercise sessions completed at the prescribed frequency, time, and intensity over 7 days/number of exercise session prescribed (as measured by accelerometry). Data from participant logbooks will be used to explain any discrepancies in the accelerometry data and to provide a similar calculation of adherence overall. To determine adherence, a threshold of 70% of sessions attended and 70% of sessions performed as prescribed will be applied.

Following 7-day accelerometry, participants will complete the International Physical Activity Questionnaire Short Form (IPAQ-SF) [41]. The IPAQ-SF contains seven items that assess physical activity over the previous 7 days. Four intensity levels of activity, including sedentary behavior, are evaluated. It is the most widely used scale to monitor physical activity, thereby facilitating comparisons between populations [42].

##### Quality of life

The Kidney Disease and Quality of Life Instrument (KDQOL-36) tool includes the 12-item Short Form Health Survey (SF-12) as generic core plus symptoms/problems of kidney disease scales [43]. The KDQOL-36 is a valid and reliable disease-specific questionnaire and has been extensively used in people with non-dialysis CKD [44]. Preference-based utilities will be measured by the EQ-5D (EuroQol Health Questionnaire) [45].

##### Adverse events

Adverse events (AEs) are defined as any unfavorable or unintended event affecting study participants. Major AEs include death, hospitalization (unplanned), emergency department visits, CV events (stroke, myocardial infarction); doubling of serum creatinine from baseline; permanent disability; syncope, arrhythmia. Minor AEs include symptomatic hypoglycemia, hyper- or hypotension requiring medical attention, musculoskeletal injury, pre-syncope during or within 1 h after exercise. Due to the low risk of the intervention we will not use a data safety and monitoring board. All serious adverse events will be forwarded to a physician who is not involved in the trial. The physician will adjudicate whether the event was due to the exercise treatment, facilitate the necessary medical follow-up, and determine whether participation should be modified.

##### Feasibility criteria

To inform the design of a future trial on the effect of exercise on the progression of CKD, we will evaluate enrollment, adherence, drop out and missing data using the following criteria: 30% of people screened are randomized [46] drop out less than 20%, missing outcome data less than 10%, and adherence to exercise sessions greater than 70% (as described above).

##### Exploratory

eGFR using the Chronic Kidney Disease Epidemiology Collaboration (CKD-EPI) equation for creatinine and cystatin C [47, 48].

### Sample size

The sample size calculations for this trial are based on data from exercise and lifestyle modification trials in hypertensive populations [49–51]. As a 5-mmHg reduction in BP in high-risk patients reduces CV events by 15% [52], this study is powered to detect a clinically important difference in mean SBP of 5 mmHg between the intervention and the control groups. Using a two-sample *t* test, alpha 0.05, beta 0.2, and assuming a common standard deviation of 10 mmHg, 128 patients are required. Assuming 20% drop out, 160 patients will be enrolled (80 per group). This sample size is also powered to detect a clinically meaningful difference of 5 points in the KDQOL-36 (alpha 0.05, beta 0.2, and assuming a common standard deviation of 10 points) [53].

### Statistical analysis

All analyses will follow the intent-to-treat principle. The difference in mean 24-h ambulatory SBP between groups at 8 weeks (primary outcome) will be analyzed using a mixed linear-regression model including fixed-effects terms for time point (8 weeks, 24 weeks), intervention, their interaction, baseline eGFR, baseline 24-h systolic ABPM, and a random-effects term for participant. In the primary analysis, missing values will be multiply imputed (100 iterations), using a hot-deck approach drawing all 8-week values from the control group with replacement, within strata of age and gender [54, 55]. Estimates from the 100 datasets will be combined using Rubin’s method [56]. Complete-case results, per-protocol and last-value-carried-forward results will be presented as sensitivity analyses. Estimates and corresponding 95% confidence intervals (CIs) will be reported for the overall difference at week 8 (and 24 weeks), adjusting for baseline eGFR and BP. All continuous outcomes with repeated measurements will be examined using mixed linear regression, following an outline similar to above. Continuous measurements without repeated measurements will be examined using linear regression, adjusting for baseline value. Residual, leverage and influence diagnostics will be examined. *P* values < 0.05 will be considered statistically significant. Adverse events will be tabulated, and compared between groups using Fisher’s exact test. All analyses will be completed in Stata/MP 15.1 (www.stata.com/).

#### Subgroup analyses

Using the model from the primary analysis, the relation between exercise and eGFR will be explored. Age and gender and corresponding treatment interactions will also be examined. Proteinuria will be considered in exploratory analyses. Changes in cardiorespiratory fitness and BMI as potential mediators of BP change will be explored.

### Data monitoring and quality assurance

Data will be entered directly at the site of data collection using tablets and an online RedCap database application. RedCap uses a secure syncing process and robust data validation techniques. The study coordinator will generate a weekly patient flow diagram reporting accrual, withdrawals, and fully completed protocols. The study coordinator will generate quality assurance logs containing information about participants’ missing or aberrant outcome testing and will then follow-up with the pertinent study personnel or participant.

## Discussion

Additional strategies to address the burden of hypertension in people with CKD are needed. Exercise is an appealing intervention due its proven efficacy in the general population and because of the potential for additional benefits. For renal healthcare providers, prescribing exercise to treat hypertension is a paradigm shift away from the mainstay of treatment, medication adjustment with or without dietary counseling on sodium intake. Therefore, to inform clinical decision-making on hypertension management in CKD, high-quality RCTs are needed to change practice. To our knowledge, this study is the first RCT designed to provide a precise estimate of the BP-lowering effect of exercise in people with moderate to severe CKD and hypertension. Strengths of this study include ABPM, which provides more accurate assessment of overall BP and kidney prognosis than clinic BPs [57, 58] and measurement of important confounders, such as anti-hypertensive dosing, medication adherence, and differences in physical activity levels between groups. Additional data on inflammation, vascular stiffness, and exercise recovery will improve our understanding of the CV response to exercise in people with hypertension and CKD.

This trial will also provide information on the effect of exercise on QoL as measured by the KDQoL. Developing care models that are more consistent with patient values and preferences and that address kidney-disease-specific symptoms (e.g., fatigue, low energy, depression) is a priority for decision-makers and patients [59, 60]. Exercise has improved several physical domains of QoL in people with CKD as measured with a generic instrument, but no trial has used a kidney-disease-specific tool [61, 62].

Towards the aim of designing a larger, multi-center trial to evaluate the effect of exercise on the progression of CKD, we will use the results from this current proposal to clarify trial processes, establish feasibility, and to estimate the variance of the treatment effect for eGFR. The need to identify lifestyle changes (e.g., exercise, reduced stress) that may delay kidney disease progression is an important question for patients [60]. The link between hypertension and renal function is well established and forms the scientific basis for first evaluating whether exercise can modify BP. Slower rates of eGFR decline were reported in a CKD cohort study comparing physically active people those who are sedentary (2.8% difference in annual eGFR decline) [63]. However, RCTs with adequate sample sizes and follow-up are needed.

## Additional file

Additional file 1: Recommendations for Interventional Trials (SPIRIT) 2013 Checklist. (DOC 120 kb)

## Abbreviations

ABPM: Ambulatory blood pressure monitoring
AE: Adverse event
BP: Blood pressure
CKD: Chronic kidney disease
CKD-EPI: Chronic Kidney Disease Epidemiology Collaboration
CPET: Cardiopulmonary exercise test
CV: Cardiovascular
DBP: Diastolic blood pressure
eGFR: Estimated glomerular filtration rate
EQ-5D: EuroQol Health Questionnaire
HgA_1_C: Hemoglobin A_1_C
KDQOL-36: The Kidney Disease and Quality of Life Instrument
PWV: Pulse-wave velocity
QoL: Quality of life
RCT: Randomized controlled trial
SBP: Systolic blood pressure
SEE scale: Self-efficacy for Exercise scale
VO_2_: Volume of oxygen consumption
